# Chilblains in Southern California: two case reports and a review of the literature

**DOI:** 10.1186/1752-1947-8-381

**Published:** 2014-11-22

**Authors:** Rebecca Gordon, Anne M Arikian, Anita S Pakula

**Affiliations:** 1 UCLA Department of Rheumatology, 2020 Santa Monica Blvd., Suite 540, Santa Monica, CA 90403, USA; 2 UCLA Department of Family Medicine Family Health Center, 1920 Colorado Avenue, Santa Monica, CA 90404, USA; 3 UCLA Division of Dermatology, 10833 Le Conte Avenue, CHS 52-121, Los Angeles, CA 90095, USA

**Keywords:** Chilblain lupus, Chilblains, Idiopathic chilblains, Pernio, Perniosis, Secondary chilblains

## Abstract

**Introduction:**

Chilblains or perniosis is an acrally located cutaneous eruption that occurs with exposure to cold. Chilblains can be classified into primary and secondary forms. The primary or idiopathic form is not associated with an underlying disease and is clinically indistinguishable from the secondary form. The secondary form is associated with an underlying condition such as connective tissue disease, monoclonal gammopathy, cryoglobulinemia, or chronic myelomonocytic leukemia. Histopathology cannot accurately help distinguish the primary from secondary forms of chilblains. This article will raise the awareness of chilblains by presenting two unusual case reports of chilblains in men from Southern California with discussion of the appropriate evaluation and treatment of this condition.

**Case presentations:**

Case 1

A 56-year-old Caucasian man presented in January to a Southern California primary care clinic with a report of tingling and burning in both feet, followed by bluish discoloration and swelling as well as blistering. He had no unusual cold exposure prior to the onset of his symptoms. He had a history of “white attacks” in his hands consistent with Raynaud’s phenomenon. His symptoms gradually resolved over a 3-week period.

Case 2

A 53-year-old Caucasian man also presented to a Southern California clinic in January with a 3-week history of painful tingling in his toes, and subsequent purplish-black discoloration of the toes in both feet. His symptoms occurred 1 week after a skiing trip. He had partial improvement with warming measures. His symptoms resolved 2 weeks after his initial presentation.

**Conclusions:**

Chilblains is a relatively uncommon entity in warmer climates but can present during the winter months. Primary care providers in warmer climates such as Southern California in the USA may be unfamiliar with its presentation. It can be diagnosed clinically by the appearance of typical lesions during the cold damp season. Through a thorough history, physical examination and selected laboratory evaluation, underlying connective tissue disease or a mimic such as vasculitis or cutaneous leukemia can be excluded.

## Introduction

Chilblains or perniosis is an acrally located eruption that occurs with exposure to cold. The lesions may be erythematous or purplish in color and may be either macular or papular. Chilblains may be primary or idiopathic, or secondary to an underlying connective tissue disease (CTD), monoclonal gammopathy or cryoglobulinemia. The differential diagnosis includes vasculitis, acrally located lesions of systemic lupus erythematosus (SLE), and emboli. A skin biopsy can help exclude these other diagnoses, but the histopathology of chilblains is nonspecific. The histopathologic findings include an inflammatory infiltrate usually extending through the dermis with associated edema, and may be concentrated in a perieccrine distribution. The epidermis may show necrotic keratinocytes. Cribier *et al.* studied biopsy specimens from patients with chilblains to determine if there are characteristic histopathologic features that distinguish primary chilblains from lupus erythematosus [1]. They concluded that it is not always possible to distinguish between idiopathic chilblains and lupus erythematosus based on histopathologic findings alone. However, lack of edema and the presence of vacuolation were more characteristic of lupus erythematosus [1].

We present two cases of chilblains from Southern California and a review of the literature. The first case is an example of secondary chilblains. The second case is an example of idiopathic chilblains.

## Case presentations

### Case 1

**Figure 1:**
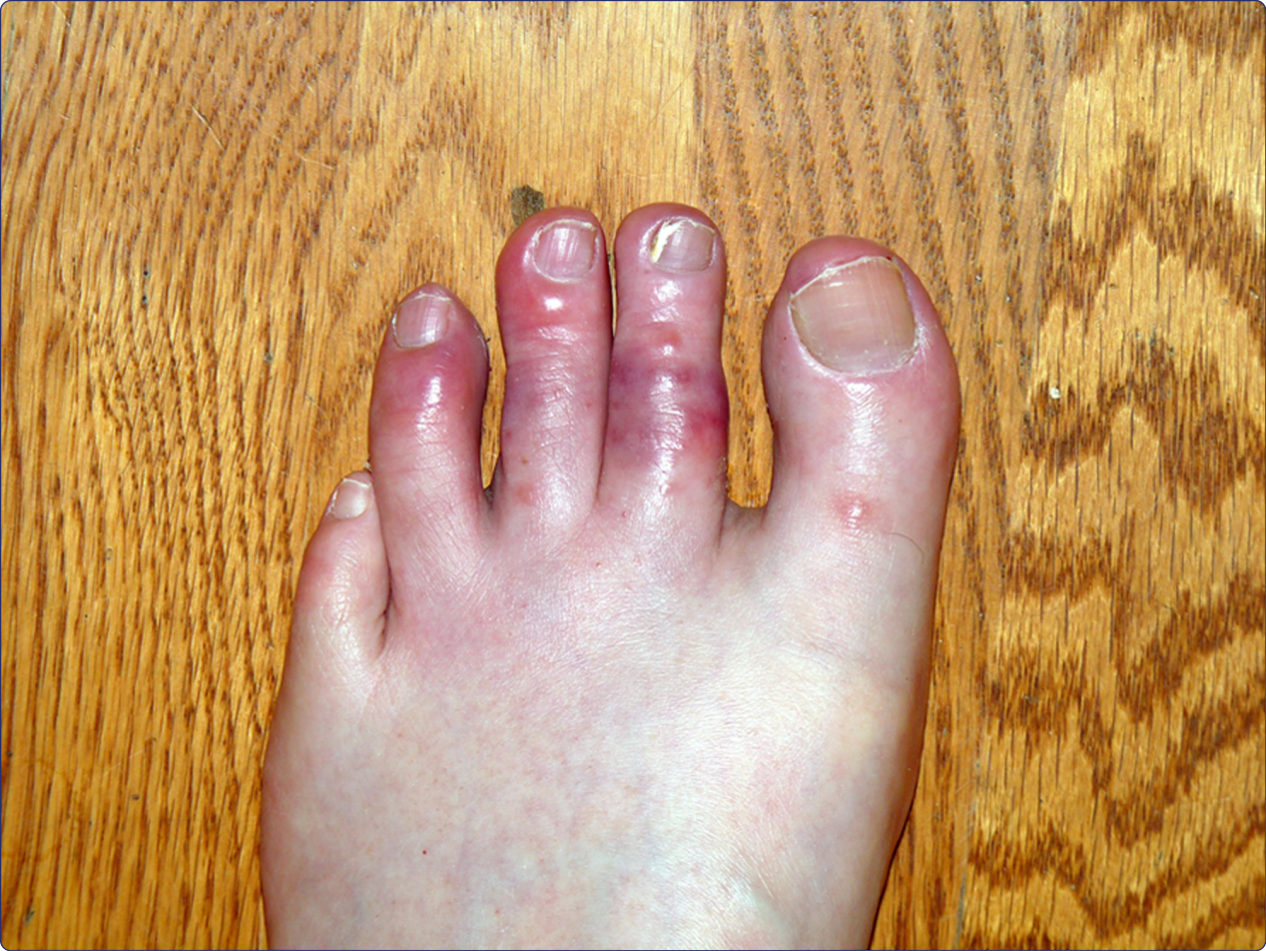
Photo taken by patient within 1 week of onset of symptoms demonstrating erythematous and purplish maculopapular lesions with vesicle formation in the toes.

A 56-year-old Caucasian man, a non-smoker, presented in late January (mid-winter) reporting that 2 weeks prior he suddenly developed tingling and burning in the toes of both feet. He then developed bluish discoloration and swelling, along with some blistering on the dorsum of his toes (Figure 1). He did not recall any precipitating events or unusual cold exposure. Over the previous 2 weeks the blistering and swelling gradually subsided and at the time of presentation only the bluish discoloration in his toes remained. Within 3 weeks his toes returned to their normal appearance.

Of note, he reports a history of “white-attacks” of the fingers upon cold exposure since early childhood (consistent with Raynaud’s phenomenon). He has no known history of CTD or autoimmune disease.

Initial laboratory findings were significant for an antinuclear antibody (ANA) positive at 1:160 ratio. He was sent to the rheumatology clinic for further evaluation. Follow-up studies included a complement component 4 (C4) level of 49 (12 to 34mg/dL), a 50% hemolytic complement (CH50) of 154 (60 to 144 CAE units), and a deoxyribonuclease (DNase-B) antibody of 232 (0 to 120U/mL). The remainder of the hypercoagulable and vasculitic work-up was negative (Table 1), as were an echocardiogram and abdominal ultrasound to rule out a source for an embolic process.

**Table 1:** Summary of laboratory data for cases 1 and 2

**Laboratory evaluation**	**Normal range**	**Case 1**	**Case 2**
Antinuclear Ab	< 1:40 titer	**1:160**	<1:40
Anti- dsDNA Ab EIA	<200IU/mL	<200	<200
Erythrocyte sedimentation rate, Westergren	0–10mm/hour	5	5
C3	71–141mg/dL	133	120
C4	12–34mg/dL	49	22
Rheumatoid factor	<25IU/mL	<10	<10
C-reactive protein	<0.8mg/dL	0.3	0.3
C-ANCA	<1:20 titer	<1:20	<1:20
P-ANCA	<1:20 titer	<1:20	<1:20
Proteinase-3 Ab	<21U	<21	<21
Myeloperoxidase Ab	<21U	<21	<21
Cardiolipin IgG	<15GPL	<15	<15
Cardiolipin IgM	<12.5MPL	<12.5	<12.5
Cardiolipin IgA	<12APL	<12	<12
Beta-2-Glycoprotein IgG	≤20SGU	<9	<9
Beta-2-Glycoprotein IgM	≤20SMU	<9	<9
Beta-2-Glycoprotein IgA	≤20SAU	<9	<9
Anti- SM Ab	<20U	<20	<20
Anti- RNP AB	<20U	<20	<20
Anti- SSA Ab	<20U	<20	<20
Anti- SSB Ab	<20U	<20	<20
CH50, Total complement	60–144 CAE units	**154**	96
Immunofixation interpretation	Normal	Normal	Normal
Cryocrit	Negative	Negative	Negative
Hepatitis B surface Ab quantitative	<10IU/L	<10	<10
Hepatitis B surface antigen	Negative	Negative	Negative
Hepatitis C Ab	Negative	Negative	Negative
Rapid plasma reagin (RPR)	Nonreactive	Nonreactive	Nonreactive

*Abbreviations:* Ab, antibody; Anti-dsDNA Ab EIA, anti-double-stranded DNA antibody by enzyme-linked immunosolvent assay; Anti-RNP Ab, antiribonucleoprotein antibodies; Anti-SM Ab, anti-Smith antibodies; Anti-SSA, anti-Sjögren’s Syndrome A antibody; Anti-SSB, anti-Sjögren’s Syndrome B antibody; C3, complement component 3; C4, complement component 4; C-ANCA, cytoplasmic antineutrophil cytoplasmic antibodies; CH50, 50% hemolytic complement; Cryocrit, percent of packed cryoglobulins; Ig, immunoglobulin; P-ANCA, perinuclear antineutrophil cytoplasmic antibodies. Bold values are abnormal.

Over a 1-year follow-up period, he experienced several episodes of Raynaud’s attacks in both his hands and feet. These episodes were characterized by red and painful digits, sometimes with swelling, but without vesicles or ulcers. He did not notice an association with the cold, but the episodes resolved within hours with warming measures.

### Case 2

A 53-year-old Caucasian man, a non-smoker who works as an auto dealer, also presenting in late January, reported that 3 weeks prior he developed painful tingling in his toes, and subsequent development of purple/black discoloration on his toes in both feet. He had been on a skiing trip approximately a week prior to the start of these symptoms. Other than some fatigue, he did not report any other symptoms, and had no other past medical history. On examination, he had flat purple/red lesions on the plantar aspects of the first through third toes, bilaterally. The lesions improved, but did not completely disappear with warming. The remainder of the examination was normal. His lesions resolved within 2 weeks of evaluation. There was no reported long-term follow up for this patient.

The results of his laboratory examination were normal, with an erythrocyte sedimentation rate (ESR) of 5. ANA, rheumatoid factor, antiphospholipid antibodies, and cryoglobulins were negative (Table 1).

An echocardiogram and abdominal ultrasound did not reveal any source of embolism.

**Figure 2:**
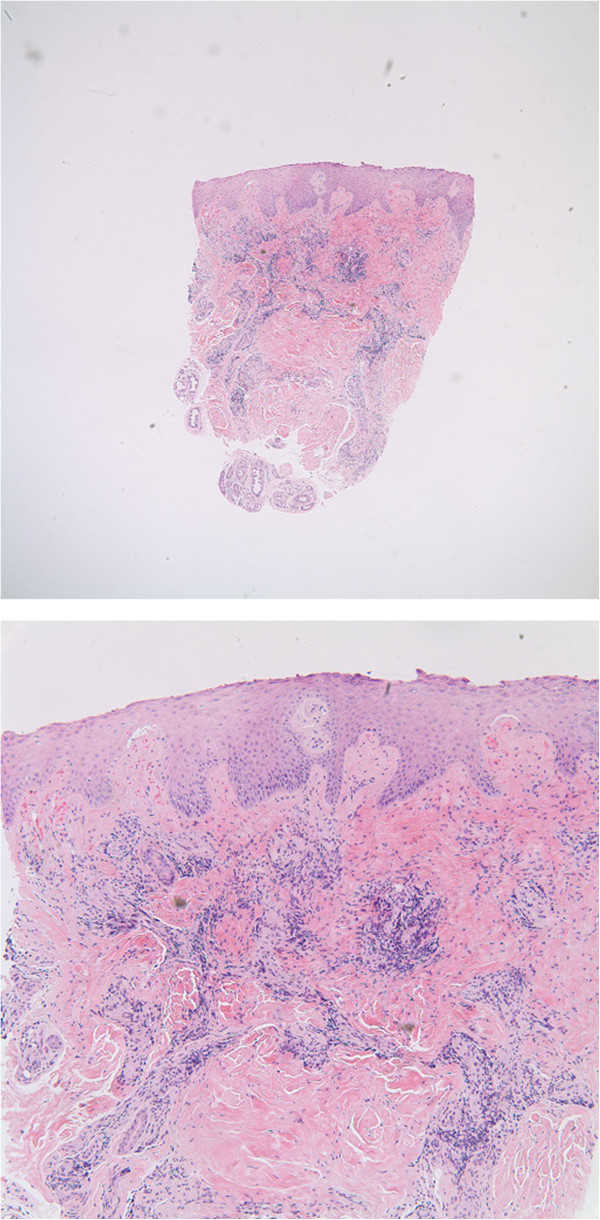
Images show a punch biopsy from the left plantar great toe with the histopathologic findings of a dense superficial and deep perivascular lymphocytic infiltrate; extravasated red blood cells are present in the upper dermis but there is no evidence of vasculitis.

Punch biopsy revealed superficial and deep perivascular lymphocytic infiltrates with focal dermal hemorrhage and mild dermal edema (Figure 2a and 2b). No vasculitis was seen. Diagnosis was consistent with chilblains/perniosis.

## Discussion

Chilblains, or perniosis, manifests as cold-induced inflammatory cutaneous lesions. It can easily be mistaken for vasculitis or an embolic event; and, as illustrated by our two cases, can be idiopathic or a secondary phenomenon related to an underlying CTD, typically SLE. A form of familial lupus chilblains exists, associated with a *TREX1* gene mutation, mostly in Asian populations [2-4].

The exact pathogenesis of chilblains is unknown. The process is generally described as a vasculopathy whereby there is disruption of normal neurovascular responses to dermal temperature changes. It has been suggested that patients with chilblains have persistent or prolonged cold-induced vasoconstriction leading to hypoxemia and a subsequent secondary inflammatory reaction [5].

There are no pathognomonic histological findings for idiopathic chilblains, but many studies describe superficial and deep perivascular lymphocytic infiltrates, with vessel wall and papillary dermal edema [1,5-7]. It has been suggested that idiopathic chilblains may be distinguished from secondary lesions seen in lupus by the presence of spongiosis, perieccrine inflammation, and dermal edema [1] in idiopathic lesions, whereas vacuolation of basal cells is more commonly seen in lupus erythematosus lesions.

Idiopathic chilblains is frequently seen in cold, damp areas of Western Europe (air humidity appears to enhance air conductivity of cold); however, an increasing number of cases are being reported in coastal areas of North America [5]. Cases typically present during the cold season of late winter to early spring. Attacks can, however, be precipitated by other types of cold exposure. Cases have been reported with use of cold therapy systems, which are used after some orthopedic procedures [8]. Lesions are almost always seen within a few hours of exposure to nonfreezing, damp conditions. Typically, they manifest as painful erythematous or purple lesions with associated swelling or itching. Overlaying erosions, ulcerations, or blisters may also be seen. Chilblain lesions are typically present symmetrically on the hands and feet. Other locations include the ear, nose, thighs, and buttocks [5]. In general, a female predominance is seen and may be associated with a low body mass index, although lesions are not uncommonly observed in men and children. Female gender is strongly associated with underlying CTD, especially SLE. The results of laboratory evaluation of idiopathic chilblains are usually normal, although an elevated ESR has been observed in some cases [5].

Secondary chilblains can present as a cutaneous feature of many conditions such as lupus (most commonly), Behcet's disease, monoclonal gammopathy, cryoglobulinemia, chronic myelomonocytic leukemia, antiphospholipid syndrome, and other CTDs. There have even been cases of tumor necrosis factor inhibitor-induced lupus chilblains [7,9]. Lesions present similarly to idiopathic chilblains, although persistence of lesions beyond the cold seasons is more common with secondary chilblains [10]. Pathogenesis is probably similar to that of idiopathic chilblains, involving an exaggerated vasoconstrictive response with subsequent hypoxemia and inflammation. However, hyperviscosity may play more of a role in the development of secondary lesions. Hypergammaglobulinemia and autoantibodies, frequently seen with CTD, may play a role in the development of these lesions, but their exact pathophysiological relevance is not known [11]. One epidemiologic study of chilblains suggests advanced age, female gender, persistence of the disorder beyond the cold season, hypergammaglobulinemia, and autoantibody positivity are suggestive of secondary chilblains [10].

The diagnosis can usually be made clinically by the appearance of typical lesions during the cold damp seasons. Idiopathic chilblains is, however, a diagnosis of exclusion, and an underlying CTD, or a mimic, such as vasculitis or cutaneous leukemia [12], must be considered. A thorough history and physical examination should be performed to evaluate for the presence of systemic disease. Initial laboratory evaluation should include: a complete blood count, ANA and antiphospholipid antibody profiles, cryoglobulins, and serum protein electrophoresis. Biopsy should be considered for treatment-resistant cases. Typical lesions in a person with a known CTD or Raynaud’s can help to make the diagnosis of secondary chilblains. Again, a biopsy may need to be considered for lesions resistant to warming measures because it is important to distinguish chilblains from vasculitis, which requires immunosuppressive therapy.

Treatment of chilblains is focused on keeping the affected area warm, and occasionally using vasodilator agents such as calcium-channel blockers. At least two small placebo-controlled studies showed rapid improvement of symptoms with use of extended-release nifedipine 20mg, taken three times daily [13,14]. Calcium-channel blockers may also play a role in prevention of recurrent or chronic lesions [7,11]. Regardless of therapy, symptoms generally resolve within days to months, although chronic, waxing and waning lesions can develop with persistent cold exposure. Secondary chilblains may have a more chronic course, and there have been cases of response to corticosteroids (systemic and topical) and mycophenolate mofetil [5,11]. Data on the use of anti-malarial medications, such as hydroxychloroquine, for both idiopathic and secondary lesions are relatively limited. Results from small retrospective studies are conflicting [15]. Some studies suggest that while hydroxychloroquine is helpful for many symptoms of CTD, it is not necessarily helpful in treating chilblain lesions [5,11].

## Conclusions

In this series, we present two cases of chilblains seen in the winter months in Southern California. One case is purely idiopathic, whereas the other is more consistent with a secondary phenomenon related to the patient’s history of Raynaud’s and an underlying undifferentiated CTD. In both cases, lesions responded well to warming measures alone. This condition needs to be recognized even in typically warmer climates, and screening for an underlying systemic disease should be done, especially in treatment-resistant cases.

## Consent

Written informed consent was obtained from the patients for publication of this case report and accompanying images. Copies of the written consents are available for review by the Editor-in-Chief of this journal.

## Competing interests

The authors declare that they have no competing interests.

## Authors' contributions

RG was a major contributor in writing the manuscript, and made the diagnosis for Case 1. AA contributed to the manuscript and interpretation of patient data, and initially evaluated the patient for Case 1. AP made the diagnosis for the patient in Case 2 and contributed to the writing of the manuscript. All authors read and approved the final manuscript.
